# Short Overview of Some Assays for the Measurement of Antioxidant Activity of Natural Products and Their Relevance in Dermatology

**DOI:** 10.3390/molecules26175301

**Published:** 2021-08-31

**Authors:** Morana Jaganjac, Vesna Sredoja Tisma, Neven Zarkovic

**Affiliations:** 1 Laboratory for Oxidative Stress, Division of Molecular Medicine Rudjer Boskovic Institute, 10000 Zagreb, Croatia; morana.jaganjac@irb.hr; 2 Polyclinic Department of Dermatology and Venereology, University Hospital Dubrava, 10000 Zagreb, Croatia; vesna.tisma@mail.inet.hr

**Keywords:** oxidative stress, antioxidants, assays, natural products, skin diseases

## Abstract

Impaired systemic redox homeostasis is implicated in the onset and development of various diseases, including skin diseases. Therefore, continuous search for natural products with antioxidant bioactivities applicable in biomedicine is attractive topic of general interest. Research efforts aiming to validate antioxidant potentials of natural products has led to the development of several assays based on various test principles. Hence, understanding the advantages and limitations of various assays is important for selection of assays useful to study antioxidant and related bioactivities of natural products of biomedical interest. This review paper gives a short overview on some chemical and cellular bioassays used to estimate the antioxidant activity of chosen natural products together with a brief overview on the use of natural products with antioxidant activities as adjuvant medicinal remedies in dermatology.

## 1. Introduction

Physical, psychological, chemical or environmental stress may provoke biological responses that can induce excessive production of reactive oxygen and nitrogen species (ROS and RNS) [1,2], which are otherwise continuously formed endogenously and contribute to the normal, oxidative energy metabolism of cells. ROS were first perceived as harmful byproducts of aerobic metabolism that may promote the onset and development of different diseases, however, their importance in redox signaling and normal cellular functioning is well recognized today. Under normal conditions generation and removal of ROS are in a fine balance. Antioxidant defense systems balance the level of prooxidants and antioxidants to maintain redox homeostasis. Disrupted redox equilibrium in favor of prooxidants will lead to oxidative/nitrosative stress. Depending on the concentration, reactivity and diffusion distance [3], ROS can react with surrounding molecules via different mechanisms, such as hydrogen abstraction and donation or acceptance of electrons [4]. The interaction of ROS/RNS with macromolecules, like proteins, carbohydrates, lipids and nucleic acids, can result in the loss or gain of function, or in the case of lipids can trigger a chain reaction of lipid peroxidation [5,6,7]. Peroxidation of lipids is of particular biological relevance as it can alter membrane fluidity, transmembrane transport and interaction of macromolecules (i.e., lipid–lipid and lipid–protein) thus impairing normal cell function or even leading to cell death [8]. Among the final products of lipid peroxidation are reactive aldehydes, such as 4-hydroxynonenal (4-HNE), which can, depending on the concentration, have a role either in the physiology or pathology of the cell [9]. In addition, reactive aldehydes are involved in various cellular processes such as regulation of cell growth, inflammation, signal transduction and apoptosis [10,11,12,13,14]. Eventually, 4-HNE can persist in the form of protein adducts being able to induce and/or propagate oxidative stress even in the absence of ROS [6,9]. Therefore, it is crucial to maintain cellular antioxidant defenses in order to avoid a rise in 4-HNE concentration from physiological to pathological levels, especially for the cells exposed to potentially harmful effects of chemical (pro-oxidants) or physical inducers (x-rays, UV-irradiation) of ROS/RNS. This is particularly relevant for skin, the largest body organ that serves as biological barrier for physical and chemical environmental factors. These factors may act as oxidants or mediators in the process of generation of ROS and RNS, and together with endogenously formed reactive species make the skin a major target for oxidative stress contributing to the onset and development of various skin pathologies. In addition, skin inflammation is associated with a number of cutaneous diseases. The formation of peroxynitrite (ONOO^−^), in the reaction between superoxide and nitric oxide (NO^•^), induces the level of nitrated proteins in the skin contributing to inflammation [15]. Furthermore, ONOO^−^ can induce lipid peroxidation yielding nitrogen-containing oxidized lipid derivatives [16] and may also induce DNA strand breakage further contributing to pathophysiology of inflammation [17].

Natural products with antioxidant bioactivity have long been recognized as a valuable tool in the management of oxidative/nitrosative stress-induced pathologies. This review discusses the natural product discovery workflow to identify products with antioxidant activity, with a focus on available chemical and cellular bioassays used to estimate the antioxidant activity. Finally, at the end of this review, special attention is given to dysregulated redox homeostasis in cutaneous diseases with a brief overview of the potential use of natural products with antioxidant activity as an adjuvant therapeutic approach in dermatology.

## 2. Synthetic Antioxidants vs. Natural Antioxidants

**Figure 1:**
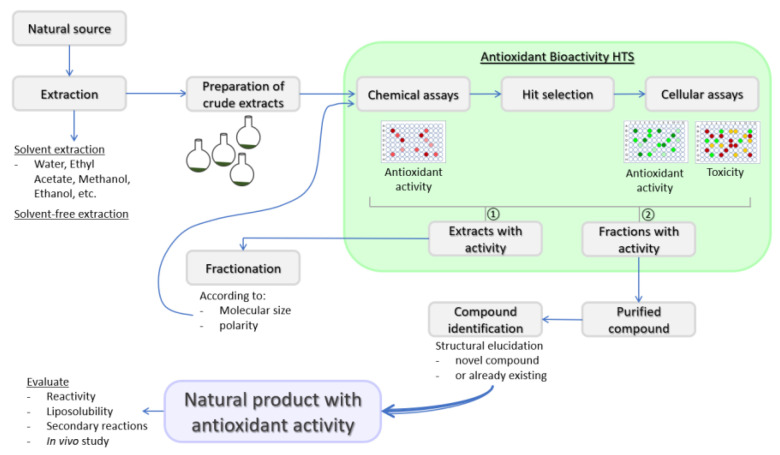
Workflow for the natural products discovery. Extracts from a natural source, such as a plant, may be prepared by either solvent extraction or solvent-free extraction. Solvent extraction is the conventional technique and the polarity index of solvents used will determine the composition of crude extracts [26]. Crude extracts are then examined for potential antioxidant properties by chemical antioxidant and only those identified as bioactive ‘hit’ extracts are further evaluated in the cellular antioxidant assays. Cellular assays also include a toxicity assay to evaluate safety. Extracts acknowledged for their potential antioxidant activity are fractionated based on specific properties in order to reduce the complexity of the extract and preferably isolate pure compounds. High throughput screening (HTS) of each fraction for the antioxidant activity is followed by the purification of compounds from fractions with promising antioxidant activity, structural elucidation and compound identification.

Substances that can delay or inhibit oxidation of a substrate, or that can upregulate antioxidant defense systems are defined as antioxidants [18,19]. Antioxidant defense systems found in humans are divided into two types: enzymatic antioxidants and non-enzymatic antioxidants. Enzymatic antioxidants are almost exclusively endogenously formed, while the origin of non-enzymatic antioxidants can be either endogenous or exogenous. The nuclear factor erythroid 2-like 2 (Nrf2), thioredoxin and glutathione (GSH) systems are among the major endogenous antioxidant defenses and their mechanisms of action for cellular ROS detoxification have been recently reviewed [20]. Exogenous antioxidants may be of natural or synthetic origin. Synthetic phenolic antioxidants (SPA) are the most frequently used synthetic antioxidants due to their low cost, higher stability and availability. Butylated hydroxytoluene, butylated hydroxyanisole and tert-butyl hydroquinone are among the most frequently used SPAs. SPAs are increasingly used globally in the food industry and consumer products and are unintended contaminants of the environment [21,22,23,24]. The emerging evidence stresses that long-term exposure to SPAs has adverse effects on human health [24], and the use of some common SPAs in the European Union has been restricted by several directives. Hence, there is a need to replace them with antioxidants of natural origin. Plants are good sources of natural antioxidants that can in general be classified as phenolic compounds, vitamins and carotenoids [25]. However, although many naturally derived compounds have antioxidant properties, the majority are not suitable for human use. Among the properties to consider are stability, active concentration and safety, just as with medicines and nutraceuticals. Thus, the evaluation of toxicity in the in vitro cellular models should be part of the natural products discovery workflow (Figure 1).

Indeed, before antioxidants can be used as food additives and/or adjuvant medicinal remedies for the integrative biomedicine purposes or as ordinary pharmacological therapeutics, their safety should be assessed by different toxicity tests as requested by the regulatory bodies, such as the European Food Safety Authority and US Food and Drug Administration [25]. Moreover, reactivity and stoichiometric factor, liposolubility and secondary reactions of new natural products with antioxidant activity should be evaluated as well [27].

## 3. Assays for the Measurement of Antioxidant Activity

**Figure 2:**
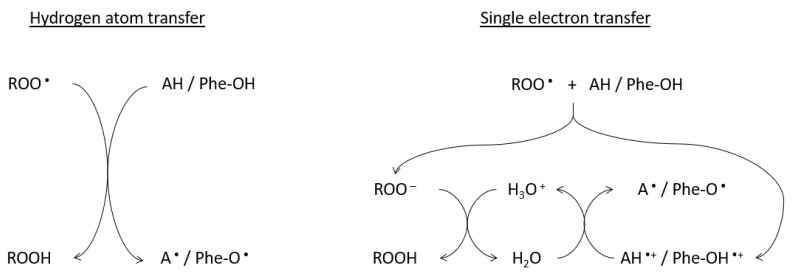
Quenching mechanisms of target radicals by hydrogen atom transfer and/or single electron transfer. AH—antioxidant; A ^•^—antioxidant radical; Phe-O ^•^—aryloxy radical; Phe-OH—phenolic compound; ROO ^•^—target radical.

Traditionally antioxidant assays are divided into hydrogen atom transfer (HAT), or single electron transfer (SET) based methods (Figure 2) [28]. Although the final products of both mechanisms might be identical, their kinetics greatly differ.

HAT assays are based on the transfer of hydrogen atoms from antioxidant or phenolic compounds to target radicals, and the kinetics of reaction depends on the solvent used. The hydrogen donation is enhanced in aqueous solutions compared to alcohol ones [29]. However, HAT assays are not dependent on pH, while pH is important for SET assays as an increase in the pH will accelerate electron transfer [30]. Some of the popular HAT-based antioxidant assays are oxygen radical absorbance capacity (ORAC), total radical-trapping antioxidant parameter (TRAP) and β-carotene bleaching assay, while commonly used SET assays include total phenolic assay, 2,3-diphenyl-1-picrylhydrazyl (DPPH) free radical method, trolox equivalent antioxidant capacity (TEAC) and ferric reducing-antioxidant power (FRAP) assay.

### 3.1. Chemical-Based Antioxidant Assays

**Figure 3:**
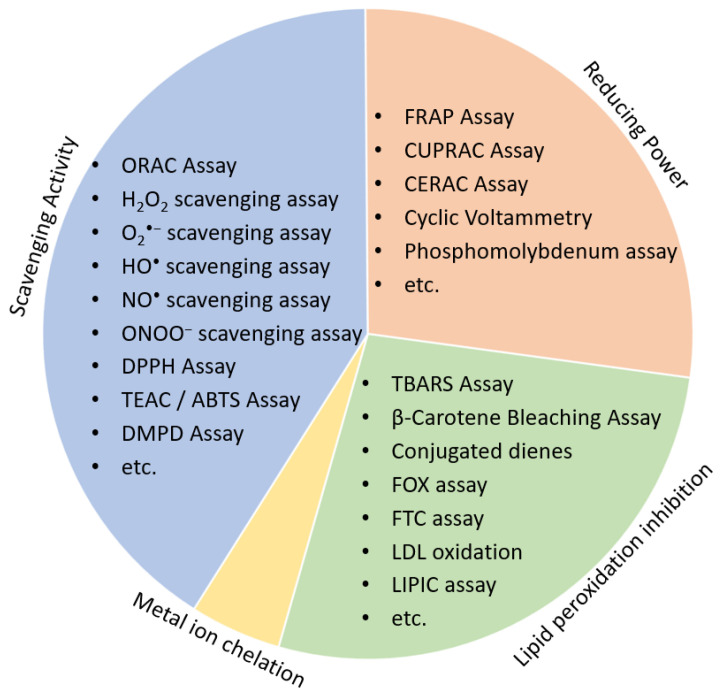
Antioxidant chemical-based assays divided based on the antioxidant activity to reducing power, lipid peroxidation inhibition, metal ion chelation and scavenging activity assay. Abbreviations: ABTS—2,2-azinobis-(3-ethylbenzothiazoline-6-sulphonic acid); CERAC—cerium reducing antioxidant capacity; CUPRAC—cupric ion reducing antioxidant capacity; DMPD—*N*,*N*-dimethyl-p-phenylene-diamine; DPPH—2,3-diphenyl-1-picrylhydrazyl; FOX—ferrous oxidation-xylenol orange; FTC—ferric thiocyanate; FRAP—ferric reducing-antioxidant power; LDL—low density lipoprotein; LIPIC—lipid peroxidation inhibition capacity; ORAC—oxygen radical absorbance capacity; TEAC—trolox equivalent antioxidant capacity; TRAP—total radical-trapping antioxidant parameter; TBARS—thiobarbituric acid reactive substances.

Low cost and high-throughput screening (HTS) assays are chemical-based methods preferred for the initial screening process of the natural products discovery workflow. Antioxidants may have different mechanisms of action, according to which antioxidant assays are divided into either (1) scavenging activity assays, (2) reducing antioxidant power assays, (3) lipid peroxidation inhibitory potential or (4) Metal ion chelation (Figure 3).

- Scavenging activity assays are based on the ability of an antioxidant to scavenge stable free radicals. Some of the most common antioxidant assays based on scavenging activity include ORAC, DPPH, 2,2′-azinobis-(3-ethylbenzothiazole-6-sulphonate) (ABTS)/TEAC assay and *N*,*N*-dimethyl-p-phenylenediamine radical scavenging (DMPD) assay [31,32]. Although the basic principle is the same, their antioxidant solubility differs from those soluble in aqueous and alcoholic media (e.g., ABTS^•+^ and DMPD^•+^ radical) to those soluble in organic solvents (e.g., DPPH^•^ radical). Other scavenging assays include those specific to certain ROS/RNS, such as ONOO^−^ [33], hydroxyl radical [34], superoxide anion (O_2_^•−^) [35] or NO^•^ scavenging assays [36].
- Reducing antioxidant power assays are based on the principle that antioxidants acts as reductants by accepting electrons, for example, from transition metals, notably iron and copper. The most frequently used reducing antioxidant power assays are FRAP, cupric ions reducing power assay (CUPRAC) and total phenolic content assay (Folin–Ciocalteu method) [32].
- The antioxidant assays widely used to evaluate antioxidant ability to inhibit lipid peroxidation include β-carotene bleaching assay, thiobarbituric acid reactive species assay (TBARS), lipid peroxidation inhibition capacity assay (LIPIC) and conjugated dienes [37,38].
- Metal ion chelation is an important property of an antioxidant as transition metals, via the Fenton reaction, promote oxidative stress and lipid peroxidation [2,39,40]. Ferrous ion is commonly used to assess the metal chelation capacity of antioxidants [41]. Curcumin, resveratrol, L-carnitine and ferrozine are among many well documented antioxidants with metal chelation ability [32].

Several comprehensive reviews on the strengths and limitations of the above chemical antioxidant assays including the detailed mechanisms behind each assay have been recently published [32,37,41,42,43].

### 3.2. Cellular-Based Antioxidant Assays

**Figure 4:**
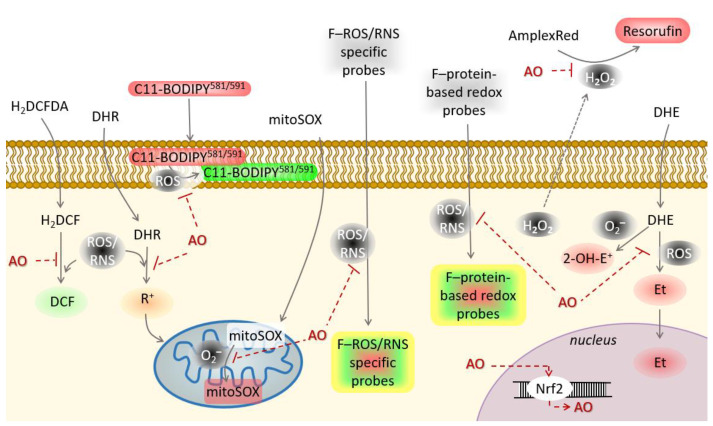
Probes used in HTS assays for the detection of ROS/RNS and lipid peroxidation. Abbreviations: 2-OH-E^+^—2-hydroxyethidium; AO—antioxidants; DCF—dichlorofluorescin; DHE—dihydroethidium; DHR—dihydrorhodamine 123; Et—ethidium; F—fluorescent; H_2_DCFDA—2′,7′-dichlorodihydrofluorescein diacetate; R^+^—rhodamine 123.

Natural products identified with potential antioxidant activity need to be further evaluated in the cellular model. Antioxidant activity of a large number of natural products will not extrapolate its performance in the biological system, either in vitro as cellular assays or in vivo as animal model studies. Thus, it is necessary to examine the bioavailability, metabolism and mechanism of action in a living system to prove potential antioxidant activities of new natural products [27]. Although in vivo studies would best reflect the effectiveness of the natural products with antioxidant activity, they are not preferred due to low throughput, occasionally bioethical uncertainties, very high costs and are time-consuming. On the other hand, in vitro cellular models, have high throughput, lower cost and are much faster. Nowadays, a plethora of redox-sensitive probes is available that enable live-cell monitoring of oxidative/nitrosative stress (Figure 4). According to the mechanism of antioxidant activity, cellular antioxidant assays could be divided into those screening for: 1. Direct reaction of antioxidants with ROS/RNS, 2. Organelle/membrane-specific antioxidant activity, 3. Inhibition of oxidant enzymes, and 4. Activators of transcription factors promoting antioxidant defense.

A variety of technologies have been developed to detect ROS/RNS, the selection of which to use will depend on several criteria, such as availability, applicability, specificity, selectivity, throughput and cost. Probes for detection of ROS/RNS in living cells are utilized to evaluate the direct effect of antioxidants on the level of reactive species. Decrease in the level of ROS/RNS can be either due to antioxidants scavenging of reactive species or due to inhibition of their generation. The majority of available probes lack specificity but can provide a good indication of potential antioxidant activity. Such probes are 2′,7′-dichlorodihydrofluorescein diacetate (H_2_DCFDA), dihdrorhodamine 123 (DHR) and chemiluminescent probes. Although initially H_2_DCFDA and DHR were perceived to detect hydrogen peroxide and ONOO^−^, respectively, it was later found that other ROS/RNS, as well as some other cellular molecules, can promote oxidation of the probes giving false-positive results [44]. Chemiluminescent assays, such as luminol or lucigenin-enhanced chemiluminescence have higher sensitivity but are susceptible to redox cycling inducing ROS formation and can, in long-term measurements, give a false increase in the signal. A number of sensitive and selective fluorescent probes for live cells/tissue detection and imaging of ONOO^−^, such as boronate-based polymeric fluorescent [45], *N*-phenylrhodol-based HKGreen-4 [46] and rhodamine based HKYellow probes [47] have been developed and could be utilized to monitor the effect of antioxidants on ONOO^−^. Dihydroethidium is another readily used probe that can react with O_2_^•−^ yielding 2-hydroxyethidium that emits red fluorescence (2-OH-E^+^). Unfortunately, DHE also nonspecifically reacts with other ROS forming ethidium, a fluorescent product whose spectra overlaps with the spectra of 2-OH-E^+^ [48]. Thus, when selecting those probes, it is crucial to be aware of all factors affecting the signal and to take precautions to reduce false-positive results.

Spin traps and spin probes used for electron-spin resonance assays have high selectivity and specificity and are among the essential tools in oxidative stress research. Similarly, redox proteomics is rapidly emerging in oxidative stress research as it can identify ROS targets and modifications induced [49,50,51]. However, low throughput and expensive instruments requiring a high level of expertise reduce their applicability. Hence, these analytical approaches could hardly be the preferred choice for HTS of new natural products.

Organelle/membrane targeted redox sensitive probes allow selection of those antioxidants with organelle/membrane specific effects. The addition of a triphenylphosphonium group to DHE led to the novel probe mitoSOX, that has specificity and selectivity for mitochondria derived O_2_^•−^ [52,53]. Afterwards, numerous organelle-targeting redox sensitive fluorescent probes were developed and validated including the redox-sensitive probes based on the chimeric fluorescent protein [54].

Screening for the antioxidant ability to protect cells from lipid peroxidation should preferably be monitored by the radiometric probe, such is C11-BODIPY^581/591^. The C11-BODIPY^581/591^ is a fatty acid analog that emits bright red fluorescence in the intact form. The probe is sensitive to ROS and when oxidized the fluorescence shifts to green [55,56].

Moreover, natural products might not act as scavengers of reactive species, but instead might inhibit activity of pro-oxidant enzymes. Among the pro-oxidant enzymes are NADPH oxidases (NOXs) and their excessive ROS production is implicated in various pathologies. A number of compounds, such as polyphenols and alkaloids from natural sources, were found to decrease or to inhibit NOX activity [57].

In addition, antioxidants could exhibit indirect activity via transcriptional regulation of Nrf2. Nrf2 is the primary redox sensor and the key regulator of endogenous antioxidant defenses, such as members of the glutathione and thioredoxin systems and superoxide dismutase (SOD) [20].

Based on all the above, two or more cellular-based antioxidant assays should be performed and interpreted in the context of data obtained from both assays to confirm the antioxidant activity of natural products.

## 4. Natural Products with Antioxidant Activity as Adjuvant Therapeutic Approach in Dermatology

Skin diseases are multifactorial, and oxidative stress plays an important role in the pathophysiology of many autoimmune and inflammatory diseases, as well as in other stress- and age-dependent diseases. Accumulating evidence shows that impaired antioxidant defenses [39,40,58,59,60] and increased levels of oxidants [39,59,60,61,62,63,64] are important mediators in the pathology of a plethora of cutaneous diseases (Table 1).

The SOD enzyme that accelerates the conversion of the O_2_^•−^ to hydrogen peroxide (H_2_O_2_), is decreased in psoriasis [65], alopecia areata [58], acne vulgaris [61], systemic sclerosis [62] and seborrheic dermatitis [59]. Alterations in the glutathione system, one of the major endogenous antioxidant defenses, were reported for alopecia areata [58], psoriasis [65], oral lichen planus [70] and atopic dermatitis [73]. In addition, vitiligo is also accompanied by decreased antioxidant defenses [67] and elevated oxidation products [68,69] that mediate the death of melanocytes [74]. Deregulated redox homeostasis and oxidation of macromolecules further accompany cutaneous disease pathology [40,61,68,71]. Nowadays, antioxidants from plants with potential antioxidant activity are emerging as potential adjuvant therapies for cutaneous diseases in order to prevent the development of various symptoms. In the Table 2 we provide a list of some natural products with antioxidant activity and with traditional, ethnomedical applications as adjuvant treatments for cutaneous diseases, or those experimentally shown in a disease model to have beneficial effects.

It is obvious that numerous plant extracts possess the capacity for medicinal applications aiming at attenuating symptoms of skin diseases, preventing their occurrence and even to be used for adjuvant therapeutic remedies or nutraceuticals, However, must follow strict regulatory rules and should be proven for their efficiency as antioxidants before being used as such.

## 5. Conclusions and Future Perspectives

While stress- and age-associated disorders are usually considered to affect internal organs, they very often manifest as skin diseases, mostly associated with skin exposure to UV light, environmental pollutants, persistent oxidative stress and chronic inflammatory processes. However, even local manifestations of the many diseases are often associated with systemic disorders of oxidative homeostasis. Therefore, if intended to be used as adjuvant medicinal remedies or even cosmetics, natural products with antioxidant potential are obliged to meet the high safety and efficacy standards, as do other medicinal remedies. They should be tested for their bioactivities using complementary assays for antioxidants. Whenever possible, antioxidant effects should be complemented by analysis of the potential pro-oxidant effects of the chosen substance(s) in the presence of biofluids (preferably serum), thus resembling complex in vivo processes, as do enzymatic assays for antioxidant capacity and peroxides [121]. Complementary to that, options for in vitro bioassays should be developed to reveal at the same time pro- and anti-oxidant activities thus resembling living systems as in the case of the 4-HNE-Cell ELISA assay, which was recently used to prove cell-type specific bioactivities of the *Aloe vera* extracts [122].

## Figures and Tables

**Table 1:** Imbalance in the redox system of cutaneous diseases.

Skin Disease	Imbalance in the Redox System	Reference
Psoriasis	Myeloperoxidase and GSH/GSSG ratio are increased	[65]
	SOD level is decreased	
Alopecia areata	SOD, paraoxonase and glutathione peroxidase are decreased	[58]
	Total antioxidant capacity is decreased	
Vitiligo	Advanced oxidation protein products, advanced glycation products and malondialdehyde levels are increased	[66,67,68,69]
	Catalase is decreased	
Rosacea	Serum peroxide and cutaneous ferritin are increased.	[39]
	Total antioxidative potential is decreased	
Acne vulgaris	Serum levels of malondialdehyde and xanthine oxidase activity are increased.	[61]
	SOD and catalase activity are decreased.	
Oral lichen planus	Salivary uric acid is decreased.	[70]
	Serum gamma glutamyl transferase (GGT) and saliva total antioxidant capacity are increased.	
Localized scleroderma (morphea)/systemic sclerosis	Total oxidant capacity, arylesterase and oxidative stress index are elevated.	[64]
	Nitric oxide, malondialdehyde, asymmetric dimethylarginine, and ROOH in the blood are elevated.	
	Levels of SOD and vitamin C are decreased.	
Chronic venous insufficiency	Malondialdehyde, serum iron and total antioxidant capacity are elevated.	[71]
	Uric acid level in the circulation is low.	
Pemphigus vulgaris	Serum bilirubin, uric acid and albumin are decreased.	[63,72]
	Serum total oxidant capacity, lipid hydroperoxides and oxidative stress index are increased.	
Eczema/dermatitis	SOD, catalase, GPX, GSH, and vitamins A, C, and E, total antioxidant status are decreased in the blood.	[59,60,73]
	Total oxidative status, total peroxides and oxidative stress index are increased.	

**Table 2:** Adjuvant therapeutic approach for the treatment of cutaneous diseases.

Skin Disease	Herbal Therapeutic Options	Mechanism of Action	References
Psoriasis	*Aloe greatheadii var. davyana*	Aqueous ethanol (95%) leaf gel extract has high polyphenol content and high antioxidant capacity.	[75]
	*Artemisia anomala S.*	Extract inactivates MAPK and caspase pathway, promotes viability of human keratinocytes and increases antioxidant capacity.	[76]
	*Astragalus sinicus* L.	Aqueous and methanol extracts possess anti-inflammatory activity, and antioxidant activity by regulating cellular redox homeostasis and NF-κB, JAK/STAT and PI3/Akt signaling pathways.	[77]
	Berry extracts	Wild blueberry, bilberry, cranberry, elderberry, raspberry seed, and strawberry possess antioxidant activity and inhibit VEGF expression and impair angiogenesis.	[78]
	Canadian wood species	Yellow birch extract and black spruce extract had highest antioxidant capacity compared to other species. Black spruce extract demonstrated low toxicity and inhibited proliferation of normal human keratinocytes and non-lesional psoriatic keratinocytes but was not selective.	[79]
	*Centella asiatica* (L.)	Polar extract modulates cyclooxygenase and lipoxygenase activities suggesting its use for the treatment of psoriasis.	[80]
	*Citrus sudachi*	Peel extract demonstrated good radical scavenging activity and high ability in reducing power. It also inhibits EGFR-ERK signaling pathway, suppressing proliferation and inducing cell differentiation.	[81]
	*Copaifera langsdorffii Desf.*	Oleoresin reduces the release of pro-inflammatory cytokines by stimulated monocytes and its treatment improved typical clinical signs.	[82]
	*Datura metel* L.	Its application significantly reduced typical clinical signs of psoriasis. It also inhibited the inflammatory response which was suggested to be due to the TLR7/8–MyD88–NF-κb–NLRP3 inflammasome pathway inhibition.	[83]
	French maritime pine bark	High antioxidant and anti-inflammatory properties by inhibiting expression of inducible intercellular adhesion molecule-1 and interferon-gamma mediated activation of Stat1.	[84]
	Indian medicinal plants	Extracts from *Phyllanthus simplex Retz., Crotolaria juncea Linn., Leucas aspera Linn*., and *Vitex glabrata R.Br.* plants inhibit NO production and lipid peroxidation in keratinocytes and have promising antiproliferative activity.	[85]
	*Melissa officinalis ssp. Altissima*	The decoction showed high free radical scavenging activity and contributed to psoriasis treatment by decreasing inflammation and enhancing barrier function.	[86]
	*Oryza sativa* L.	Crude extract has an antioxidative property by enhancing Nrf2, induces expression of anti-inflammatory cytokines while reduces proinflammatory cytokines, impairs expression of psoriasis-associated genes and improves typical clinical signs of disease.	[87]
	*Plectranthus madagascariensis*	Contains abietane diterpenoids with excellent antioxidant activity.	[88]
	*Solanum xanthocarpum Schrad. & Wendl.*	Ethanolic stem extract has antioxidant properties and was found to inhibit the expression of proinflammatory cytokines and improves typical clinical signs of psoriasis.	[89]
Alopecia areata	Ginger *(Zingiber officinale (L.) Rosc)*	Orally administered ginger powder elevated GSH level and reduced malondialdehyde level of erythrocytes and lymphocytes, and improved total antioxidant status in alopecia areata patients	[90]
	Herbal extract	Extract prepared from *Urtica dioica* root, *Urtica urens* Leaf, *Equisetum arvense* leaf, *Achillea millefolium* aerial part, *Matricaria chamomilla* flower and *Ceratonia siliqua* fruit with known antioxidant and anti-inflammatory properties, downregulates expression of IL-1alpha a mediator for the hair loss.	[91]
Vitiligo	*Clusia minor* L.	Extract of this plant used to treat vitiligo exhibit antioxidant activity as determined by radical scavenging activity and ferro-reducing activity.	[92]
	Date seed	Date seed oil has radical scavenging activity, inhibits lipid peroxidation and protects against H_2_O_2_-induced cell death of melanocytes.	[93]
	Ginger	An active compound 6-shogaol has protective effects against H_2_O_2_-induced cell stress and activates Nrf2 pathway in epidermal melanocytes.	[94]
	*Ginko biloba*	Terpenoid bilobalide protects melanocytes from H_2_O_2_-induced apoptosis, promotes catalase and glutathione peroxidase 1. Bilobalide also exhibited immunoprotective effect by reducing the release of Hsp70.	[95]
	Green tea	Protects melanocytes from H_2_O_2_-induced cell death. Among the major constituents of green tea is Epigallocatechin-3-gallate with high antioxidant and anti-inflammatory potential that was also found to inhibit Janus kinase 2 thus suppressing trafficking of T lymphocytes to melanocytes.	[96,97]
	*Pyrostegia venusta*	Topical and oral administration of leaves extract has antioxidative and anti-inflammatory properties and increases epidermal melanin level in and animal vitiligo model.	[98]
	*Scutellaria baicalensis*	Baicalein extracted from the plant protects melanocytes from H_2_O_2_-induced apoptosis and promotes activation of Nrf2 pathway.	[99]
	*Vernonia anthelmintica (L.) Willd.*	The extract contains compounds with antioxidant properties and promotes melanogenesis.	[100]
Rosacea	Turmeric *(Curcuma longa)*	Turmeric has antioxidant and anti-inflammatory properties and the administration of turmeric polyherbal formulation reduces facial redness intensity and distribution.	[101,102]
Acne vulgaris	*Artemisia vulgaris*	Essential oil has antioxidant properties with strong metal chelation activity and inhibits growth of *Streptococcus pyogenes* and *Propionibacterium acnes*.	[103]
	*Cephalaria uralensis*	Ethanolic extract of aerial parts demonstrated radical scavenging activity, inhibits cyclooxygenase-1 and -2, and inhibits growth of *Staphylococcus aureus*, *Staphylococcus epidermidis*, and *Propionibacterium acnes*.	[104]
	*Clausena anisata*	Extract inhibits growth of *Propionibacterium acnes*, has potent antioxidant activity, inhibits lipase and hyaluronidase activity and decreases IL-8 production.	[105]
	*Helichryssum kraussii*	Extract inhibits growth of *Propionibacterium acnes*, has potent antioxidant activity	[105]
	*Humulus lupulus* L.	Hop extracts demonstrated antibacterial activity against five acne causing bacteria, anticollagenase inhibitory activity and good antioxidant capacity.	[106]
	Keishibukuryogan-ka-yokuinin (KBGY)	Oral administration inhibits formation of lipid hydroperoxides and scavenges ROS in plasma.	[107]
	*Mangifera indica* L.	Kernel extract inhibits growth of *Propionibacterium acnes*, has strong radical scavenging properties, inhibits linoleic acid peroxidation and secretion of IL-8.	[108]
	*Neolitsea aciculata*	Essential oil inhibits growth of *Propionibacterium acnes* and *Staphylococcus epidermidis*, has antioxidant properties and reduces release of inflammatory cytokines.	[109]
	*Origanum vulgare*	Ethanolic extract reduces generation of inflammatory cytokines and suppresses *Propionibacterium acnes* induced skin inflammation	[110]
	*Sargassum polycystum C. Agardh*	Methanolic fractions inhibit growth of *Propionibacterium acnes*, inhibits lipase and have high radical scavenging activities.	[111]
	*Selaginella involvens*	The extract inhibits production of NO, has NO scavenging effect and inhibits growth of *Propionibacterium acnes*.	[112]
	*Syzygium jambos* L.	The ethanol extract inhibits the growth of *Propionibacterium acnes*, exhibits strong antioxidant activity and inhibits the release of inflammatory cytokines.	[113]
Oral lichen planus	Neem tree	Mouthwash with aqueous neem leaves extract improved symptoms of disease in patients.	[114]
	Purslane	Oral administration of antioxidant-rich purslane led to partial or complete clinical improvement in majority of patients.	[115]
Chronic venous insufficiency	Red-vine-leaf	Extract AS195 induces activation of endothelial and red blood cell nitric oxide synthase increasing NO bioavailability and ameliorates tert-butylhydroperoxide induced ROS.	[116]
	*Ruscus aculeatus*	*Ruscus* extract showed significant venular constriction, antioxidative and anti-inflammatory properties.	[117]
Eczema/ dermatitis	*Erythrina stricta Roxb.*	*Erythrina* extracts are active against *Staphylococcus aureus* and *Candida albicans*, and extracted erynone demonstrated significant radical scavenging activity.	[118]
	*Sapium sebiferum (L.) Roxb.*	Phenolic extracts from leaves increase activities of catalase and SOD, increase GSH level and exhibit anti-inflammatory properties in an animal dermatitis model.	[119]
	*Sophora alopecuroides* L.	The root extract has strongest antioxidant activity and showed inhibitory activity for different enzymes.	[120]
